# Assessment of Functional and Aesthetic Results in Preservation and Structural Rhinoplasty

**DOI:** 10.3390/jcm15041429

**Published:** 2026-02-12

**Authors:** Sandra Krzywdzińska, Dariusz Jurkiewicz, Marcin Jadczak

**Affiliations:** Department of Otolaryngology with Division of Cranio-Maxillo-Facial Surgery, Military Institute of Medicine—National Research Institute, 04-141 Warsaw, Polandmjadczak@wim.mil.pl (M.J.)

**Keywords:** preservation rhinoplasty, structural rhinoplasty, rhinoplasty

## Abstract

**Background:** The philosophy of preservation rhinoplasty (PR) is gaining popularity as an alternative to structural rhinoplasty (SR), prompting comparison of their effectiveness, patient satisfaction, and impact on quality of life. **Methods:** This study evaluated the outcomes of rhinoplasty performed using PR and SR, supported by the SCHNOS, ROE, NOSE, and WHOQOL-BREF questionnaires. Seventy-five patients were included (N = 47 (63%) female and N = 28 (37%) male), of whom 39 underwent SR. Surveys were completed preoperatively and at one and three months postoperatively. A single surgeon performed all procedures, and the results were subjected to statistical analysis. **Results:** Baseline ROE scores were identical in both groups (1.2 vs. 1.2; *p* > 0.05). At three months, PR demonstrated significantly higher ROE scores (3.8 vs. 3.7; *p* < 0.05). In the SCHNOS-Obstruction domain, PR showed better outcomes at one month (0.8 vs. 1.3; *p* < 0.01), whereas three-month scores were equal (0.5 vs. 0.5; *p* > 0.05). SCHNOS-Cosmesis and NOSE outcomes did not differ significantly at any point. WHOQOL-BREF results indicated significant postoperative improvement across all domains in both groups (*p* < 0.001). **Conclusions:** PR provides higher early satisfaction, but both PR and SR yield progressive improvement and comparable longer-term outcomes. Quality of life and self-esteem improved regardless of surgical technique.

## 1. Introduction

The proportions and shape of the nose, as well as prevailing aesthetic standards, have changed over the years, reflected in the development of nasal surgery techniques. Rhinoplasty is a procedure that simultaneously improves nasal patency through septal correction and external nasal shape. In recent years, preservation rhinoplasty (PR) has been gaining popularity as an alternative to classic structural rhinoplasty (SR).

PR involves minimal tissue resection and preservation of ligaments, cartilage, and the natural lines of the nasal bridge, which helps achieve predictable aesthetic and functional outcomes with less surgical intervention. Unlike SR, which often requires significant reconstruction of the nasal structures, PR aims to model them while preserving their anatomical integrity. Comparing PR and SR is therefore crucial for assessing postoperative outcomes, recovery time, and the risk of complications and enables an informed choice of surgical method tailored to the individual needs of the patient.

The study aimed to compare the treatment outcomes of patients after SR and PR and to evaluate the treatment outcomes using the Standardised Cosmesis and Health Nasal Outcomes Survey (SCHNOS), the Rhinoplasty Outcome Evaluation (ROE), and the Nasal Obstruction Symptom Evaluation (NOSE) after surgical treatment in relation to the preoperative status, as well as to assess the improvement in quality of life using the WHO Quality of Life (WHOQOL-BREF) in patients after surgical treatment.

## 2. Materials and Methods

The study included 75 participants [N = 47 (63%) females and N = 28 (37%) males] aged between 18 and 63 (M = 34; SD = 11) who qualified for rhinoplasty. SR was performed in 39 individuals [N = 26 (67%) women and N = 13 (33%) men] aged 18 to 62 (M = 34; SD = 10). PR was performed in 36 individuals [N = 21 (58%) women and N = 15 (42%) men] aged 18 to 63 (M = 34; SD = 11). The same surgeon (with over 25 years of experience in rhinoplasty and highly proficient in all techniques used) performed all of the operations. Patients were provided with oral and written information. Informed consent was obtained from all individual study participants. The study was approved by the Bioethics Committee of the Military Medical Chamber, Resolution No. KB [39/23] and KB [24/24]. We confirm that written informed consent for publication was obtained from all patients whose photographs are included in the manuscript. The consent explicitly covers the processing and publication of clinical images for scientific purposes.

The study was conducted on patients who met the inclusion criteria: age over 18, presence of a nasal hump, no history of paranasal sinus disease, depression or body dysmorphic disorder (BDD), and the necessity for rhinoplasty. An additional criterion for inclusion in the study was the finding that the patient could have undergone either conventional surgery or preservation rhinoplasty successfully, with no apparent advantage of either method.

The exclusion criteria included status post rhinoplasty, status post cleft lip and palate, status post orthognathic surgery, significant curvature of the nasal pyramid accompanied by complex curvature of the nasal septum, excessively wide nasal bridge, and the presumed potential to achieve greater postoperative benefits with a specific surgical technique. Participants who did not consent to participate in the study, opted out of the follow-up, or refused to complete the questionnaires were excluded from the study.

All patients who qualified for surgery had an extensive medical history taken and underwent a thorough preoperative assessment, a medical examination, and surgical planning. Preoperative photographs were taken and computer visualisations were created.

The study assessed the skin, the shape of the nose and its alignment with the face, and the internal structures of the nose. The photographs and visualisations included frontal, lateral, base, oblique, and dorsal views (also known as the helicopter view). The problem and the objectives of the procedure were discussed with each patient individually in an easy-to-understand manner.

Both PR and SR surgeries were performed under general endotracheal anaesthesia using an extranasal approach in the shape of an inverted “V”.

During surgery, elements of structural rhinoplasty were employed, including lowering of the bony nasal dorsum using an osteotome or piezoelectric device, bilateral percutaneous transverse osteotomies, and intranasal lateral and intermediate osteotomies. The middle third of the nose was reconstructed using either a spreader flap or spreader graft, depending on the clinical case. In every case, the patient was informed preoperatively about the surgical plan and potential intraoperative modifications.

During PR, septal reduction techniques were performed using either a high strip or a low strip—along with removal of a fragment of the perpendicular plate of the ethmoid bone, excision of the Webster triangle, application of the ballerina manoeuvre, the let-down technique, and lateral and transverse osteotomies. Reconstruction of the middle third of the nose was, by definition, not required. In PR, ligament reconstruction was performed at a later stage to achieve the best possible postoperative outcomes.

After the procedure, both groups received septum-stabilising plates, bilateral anterior packing, and an external thermoplastic nasal splint. The anterior packing was removed 24 h after the procedure. Patients were advised to clean their nasal cavities with seawater preparations. The external dressing, skin sutures, and septum-stabilising plates (silicone (Silastic) splints) were removed 7 days after the procedure.

In the 75 cases included in this study, there were no major complications such as hematoma, infection, significant postoperative irregularities or need for revision surgery. Minor complications did occur in a few cases, including slight bleeding on the second postoperative day and mild bruising, which resolved spontaneously without intervention.

Patients were given questionnaires before (1st measurement) and after surgery [respectively, one month (2nd measurement) and three months (3rd measurement)], comprising the standardised SCHNOS, ROE, and NOSE. In addition, patients completed the WHOQOL-BREF before and after surgery.

SCHNOS allows assessment of functional and aesthetic outcomes in patients after rhinoplasty. The questionnaire consists of two domains: aesthetic (SCHNOS-Cosmesis, SCHNOS-C) and functional (SCHNOS-Obstruction, SCHNOS-O). SCHNOS-C contains 6 items, including “Decreased mood and self-esteem due to my nose”, “The shape of my nasal tip”, “The straightness of my nose”, “The shape of my nose from the side, “How well my nose suits my face” and “The overall symmetry of my nose”. SCHNOS-O includes 4 items: “Having a blocked or obstructed nose”, “Getting air through my nose during exercise”, “Having a congested nose” and “Breathing through my nose during sleep”. In total, the questionnaire contains 10 items. Responses are given using a Likert scale (from 0 to 5). A score of 5 is interpreted as an “extreme problem”, while 0 means “no problem”. In short, the lower the score, the greater the patient satisfaction. The maximum score possible on the questionnaire is 50 and the lowest is 0 [1].

The ROE questionnaire is an easy-to-use tool developed in 2001 by Alsarraf [2] to assess satisfaction and outcomes of rhinoplasty. It consists of six questions assessing physical issues (based on patient satisfaction with the shape and function of their noses), emotional issues (as measured by self-confidence and willingness to change their appearance) and social factors (based on social, professional, and family acceptance). Each question has five possible answers, rated from zero to four. The maximum possible score for a patient is 24 and the lowest is 0. In the case of the ROE questionnaire, unlike SCHNOS, higher scores indicate greater patient satisfaction. Since its inception, the ROE questionnaire has been translated into many languages and popularised as an effective method of assessing postoperative outcomes [3,4].

**Figure 1:**
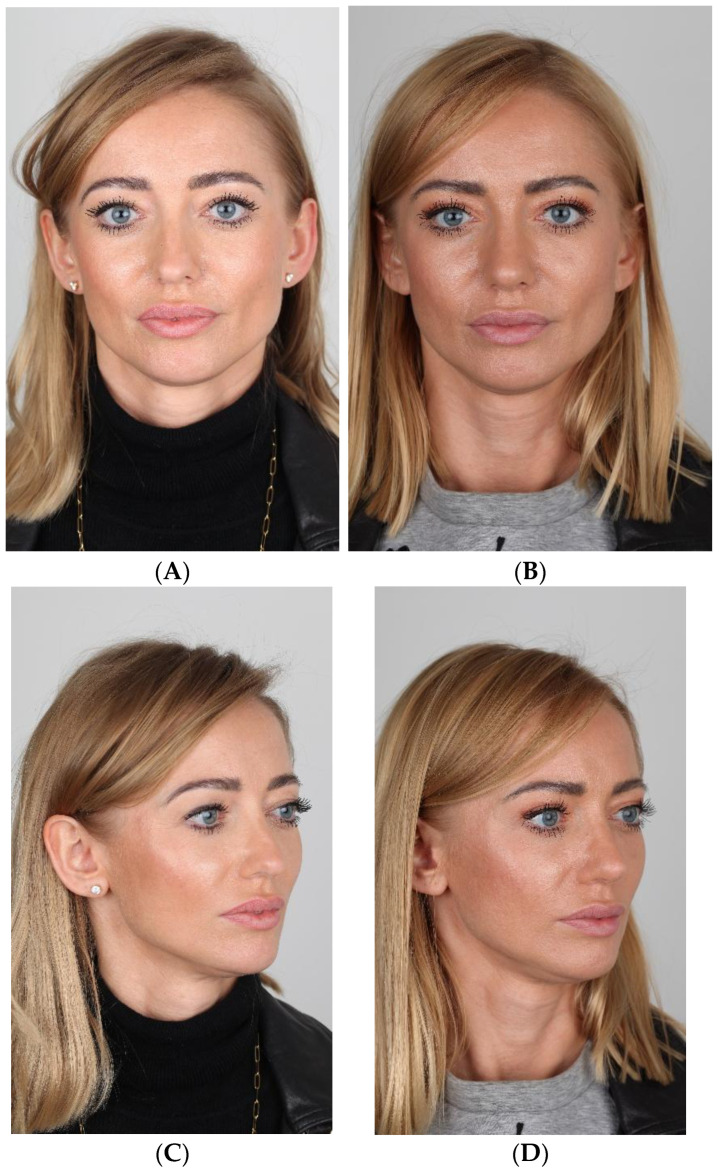

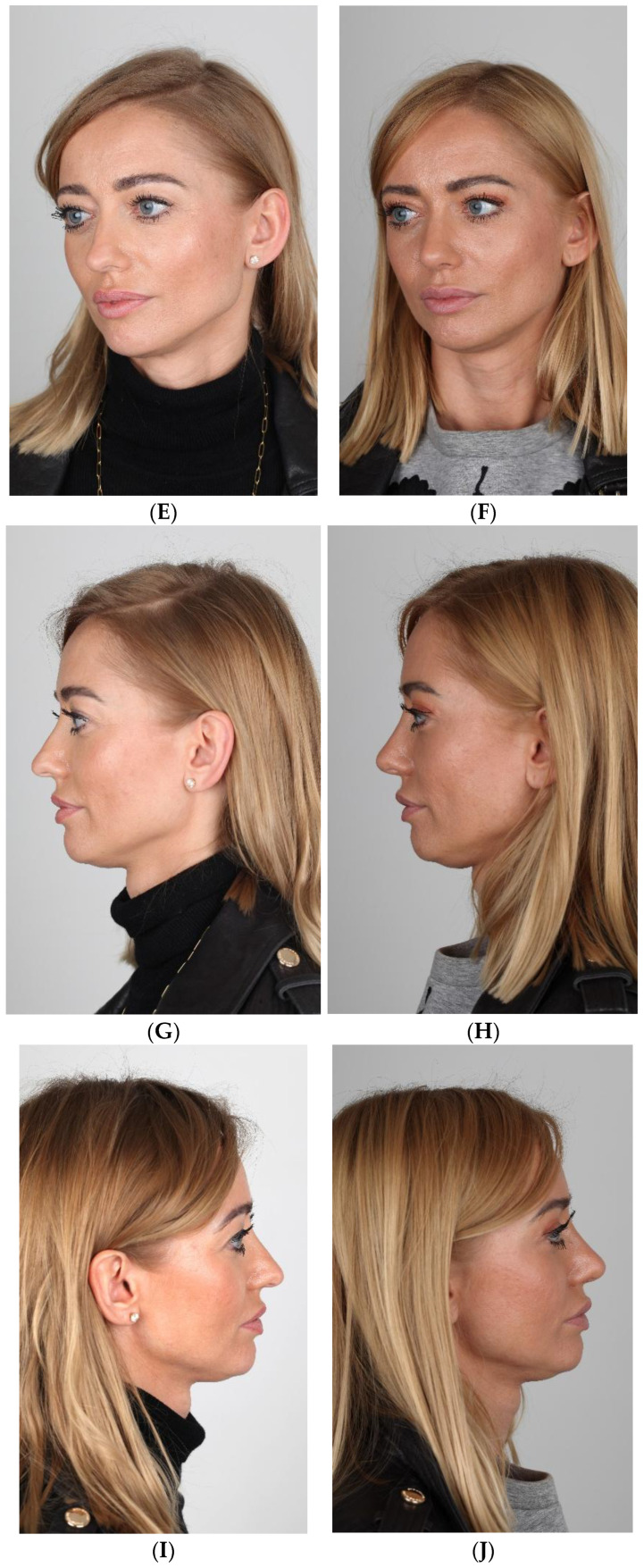
(**A**–**J**) Pre- (**A**,**C**,**E**,**G**,**I**) and postoperative (**B**,**D**,**F**,**H**,**J**) pictures of patient (PR).

Stewart et al. [5] developed the NOSE questionnaire in 2004 to assess nasal obstruction symptoms. It consists of 5 questions assessing the impact of nasal obstruction over the past 30 days on the patient’s life [5,6,7]. The questions cover nasal congestion, nasal blockage or obstruction, trouble breathing through the nose, sleep problems caused by nasal obstruction, and difficulty getting enough air through the nose during exercise or exertion (Figure 1). As with the ROE, each question has five possible answers, rated from zero to four. Where 0 means “not a problem” and 4 is considered a “severe problem”. In the case of the NOSE questionnaire, as in SCHNOS and unlike ROE, the lower the score, the greater the patient satisfaction [8].

The WHOQOL-BREF is a tool for assessing quality of life for clinical purposes [9]. It is a shortened version of the WHOQOL-100, developed in the early 1990s for the WHO as a universal research tool for assessing quality of life. It contains 26 questions for analysing four domains and, separately, the overall quality of life and self-assessment of health [10].

The data were statistically analysed and compared between groups.

### Data Analysis

Statistical analyses were performed using IBM SPSS Statistics 29.0. The program was used to calculate basic descriptive statistics and to perform the Shapiro–Wilk test to assess the distribution’s conformity with the normal distribution. The Mann–Whitney U test was used to compare the results of the two groups. For comparing two measurements, the Wilcoxon test was used; for comparing more than two measurements, the Friedman test was used. Dunn’s test with Bonferroni significance level correction was used as a post hoc test.

For variables meeting the assumptions of normality, homogeneity of variance, and sphericity, an analysis of variance was performed in a 2 × 2 mixed design. The Bonferroni test was used as a post hoc test. Pearson’s r and Spearman’s rho correlation coefficients were used to establish the relationship between the variables. For the purposes of this report, α = 0.05 was adopted as the level of significance.

## 3. Results

Table 1 and Table 2 present descriptive statistics for the questionnaire results in the analysed patient groups.

The Shapiro–Wilk test showed that the following variables were normally distributed:

(a) In patients after PR:
  a. for data in the first measurement (preoperative assessment): ROE; SCHNOS-O; quality of life in the somatic, psychological, and environmental domains; self-assessment; and NOSE
  b. for data in the second measurement (assessment one month after surgery): SCHNOS-O and quality of life in the psychological domain
(b) in patients after SR:
  a. for data in the first measurement (preoperative assessment): ROE; SCHNOS-O; Total SCHNOS; quality of life in the somatic, psychological, and environmental domains; and NOSE
  b. for data in the second measurement (assessment one month after surgery): SCHNOS-O and quality of life in the psychological domain

For the remaining variables in both groups, distributions deviating from normality were observed. Nevertheless, the skewness values for dimensions related to quality of life assessment and self-assessment did not exceed an absolute value of 1, indicating that the deviation was not significant (George and Mallery, 2019) [11]. For ROE and SCHNOS measurements, skewness values were relatively high, so analyses were performed using non-parametric tests.

### 3.1. Comparison of Postoperative Patient Groups (PR) and (SR) Regarding ROE, SCHNOS, and NOSE

Table 3 presents a comparative analysis of postoperative PR and SR patients regarding ROE < SCHNOS and NOSE. The analysis revealed statistically significant differences between the groups for ROE measurement 3 and SCHNOS-O measurement 2. This indicates that patients who had classic surgery (SR) had statistically significantly lower ROE scores in measurement 3 (weak effect) but statistically significantly higher SCHNOS-O scores in measurement 2 (moderate effect) compared with patients who underwent PR. For the other parameters, there were no differences between patients.

### 3.2. Comparison of Questionnaire Results Between Measurements in Patients After PR and SR

To determine differences between the ROE and SCHNOS questionnaire measurements, a Friedman test was performed. The analysis showed statistically significant differences between measurements for all analysed variables in both patient groups. Post hoc analysis indicated that patients achieved statistically significantly lower ROE scores in the first measurement than in the other two measurements. This was true for both patient groups. For SCHNOS-O and Total SCHNOS, the analysis showed statistically significant differences across all measurements—the highest scores were obtained in the first measurement, while the lowest were in the last. For SCHNOS-C, the analysis showed a statistically significantly higher score in the first measurement than in the other two measurements. These results are replicated in both patient groups. Detailed analysis results are summarised in Table 4.

Table 5 presents the results of the Wilcoxon test for NOSE.

It was shown that the first measurement yielded statistically significantly higher results than the second measurement. Similar results were observed in both groups. The effect size for the differences was strong.

### 3.3. Comparison of Groups in Terms of Quality of Life Assessment and Self-Assessment

In order to compare groups of patients operated on using the PR and SR methods in terms of quality of life assessment and self-assessment in two measurements, a mixed-design analysis of variance was performed, where the between-subjects factor was the type of surgery and the within-subjects factor was the repeated measurement of quality of life assessment and self-assessment. The models took into account the interaction of effects. Detailed results of the analyses are presented in Table 6.

### 3.4. Pictures of Patients

**Figure 2:**
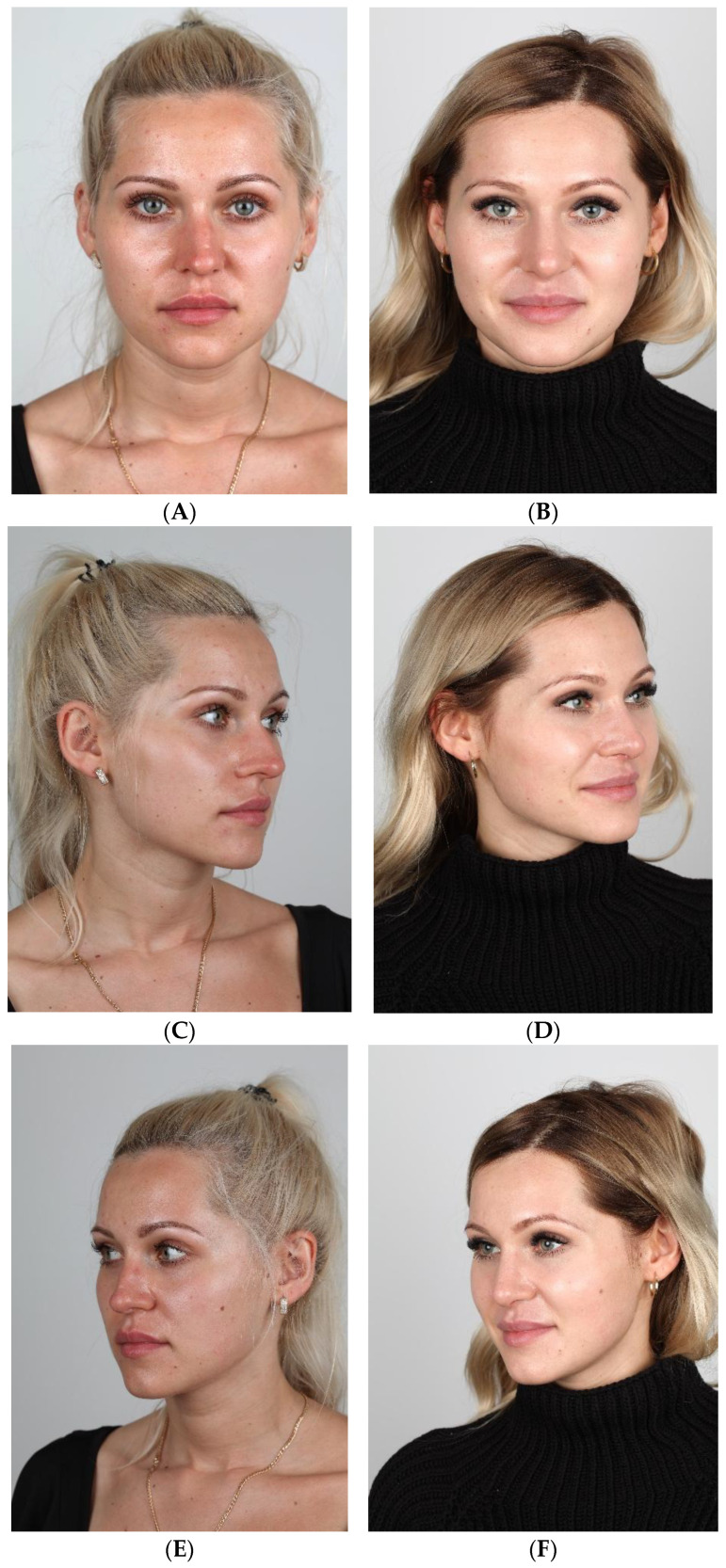

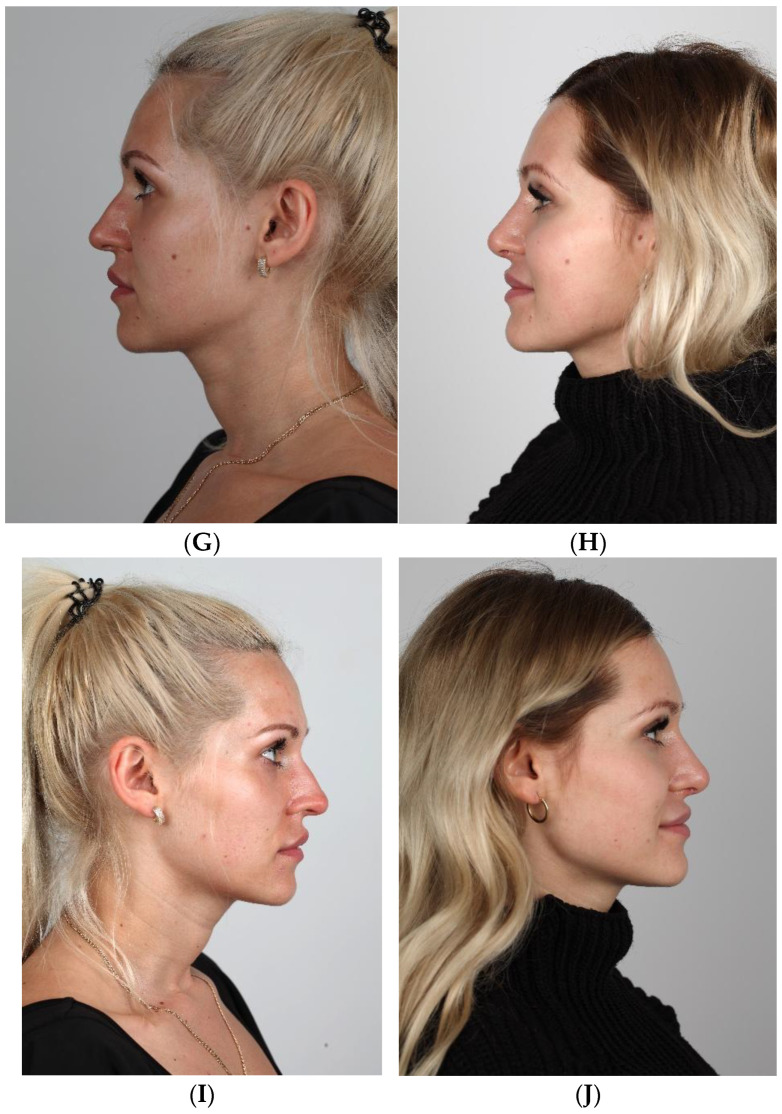
(**A**–**J**) Pre- (**A**,**C**,**E**,**G**,**I**) and postoperative (**B**,**D**,**F**,**H**,**J**) pictures of patient (SR).

**Figure 3:**
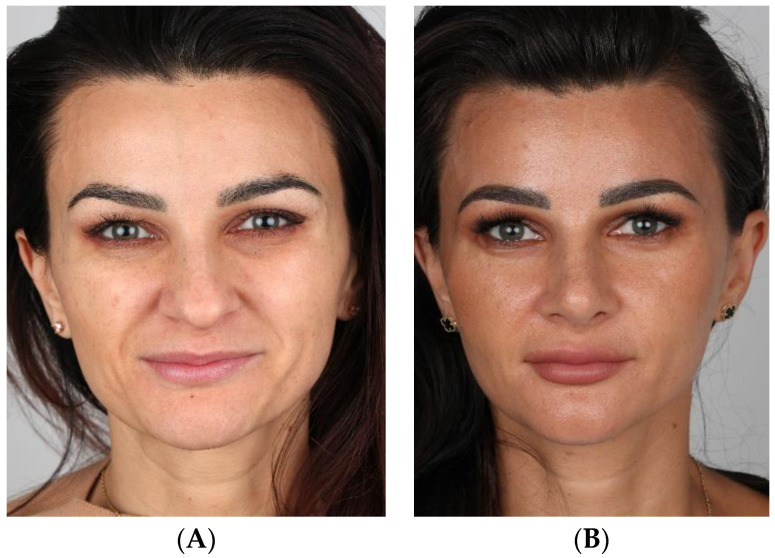

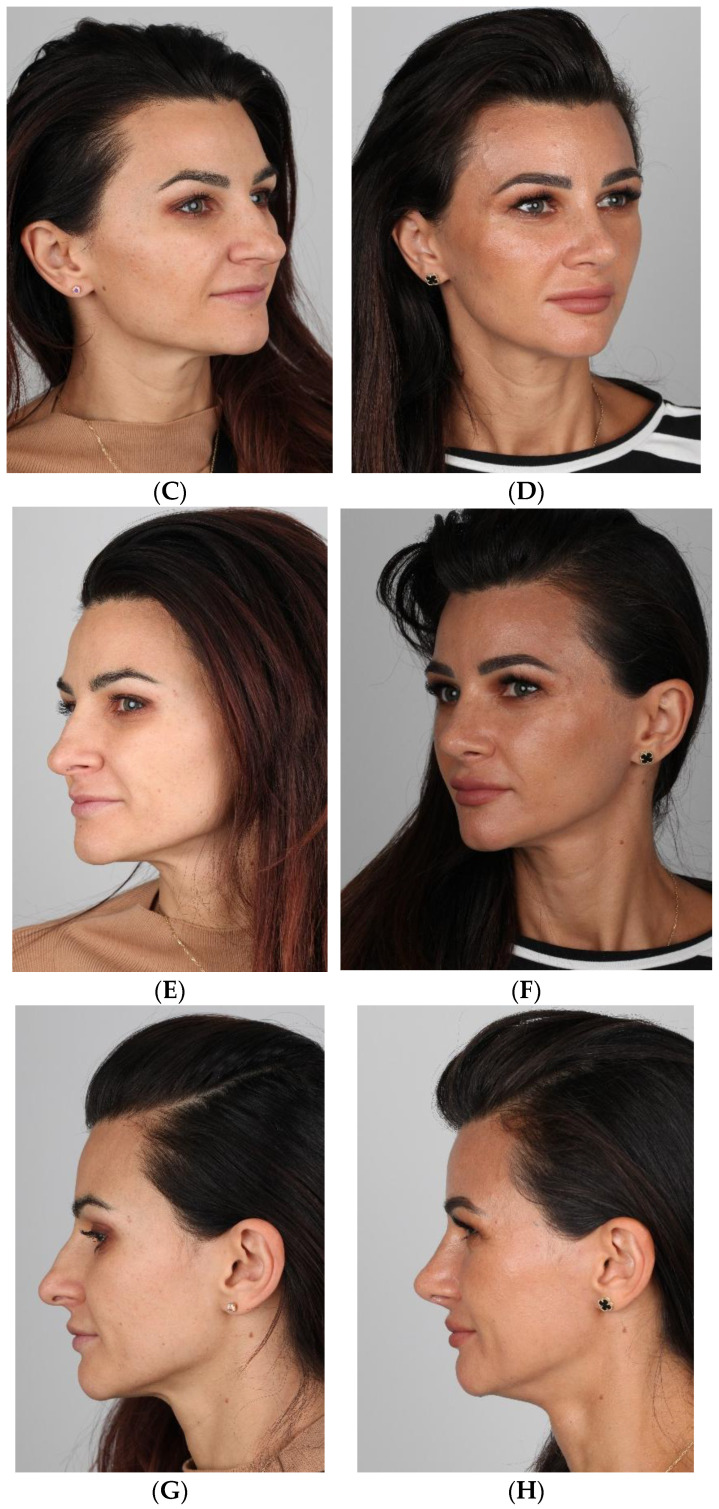

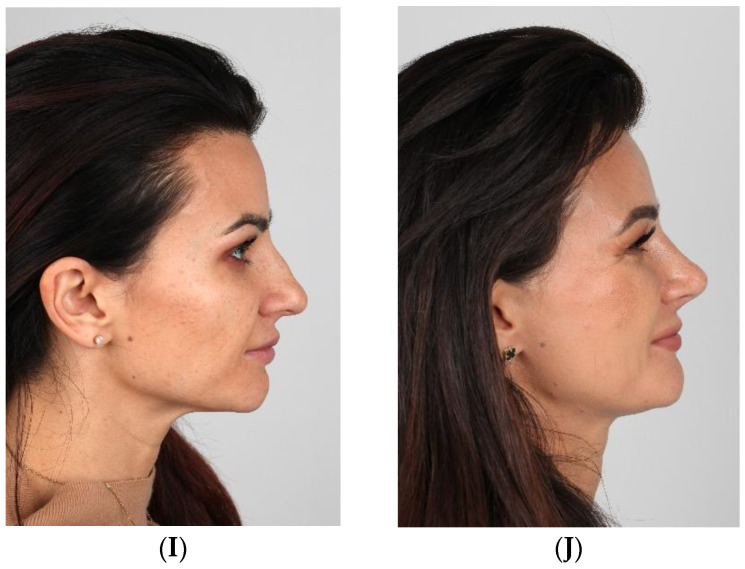
(**A**–**J**) Pre- (**A**,**C**,**E**,**G**,**I**) and postoperative (**B**,**D**,**F**,**H**,**J**) pictures of patient (SR).

Figure 2A–J and Figure 3A–J show cases treated using the SR technique.

**Figure 4:**
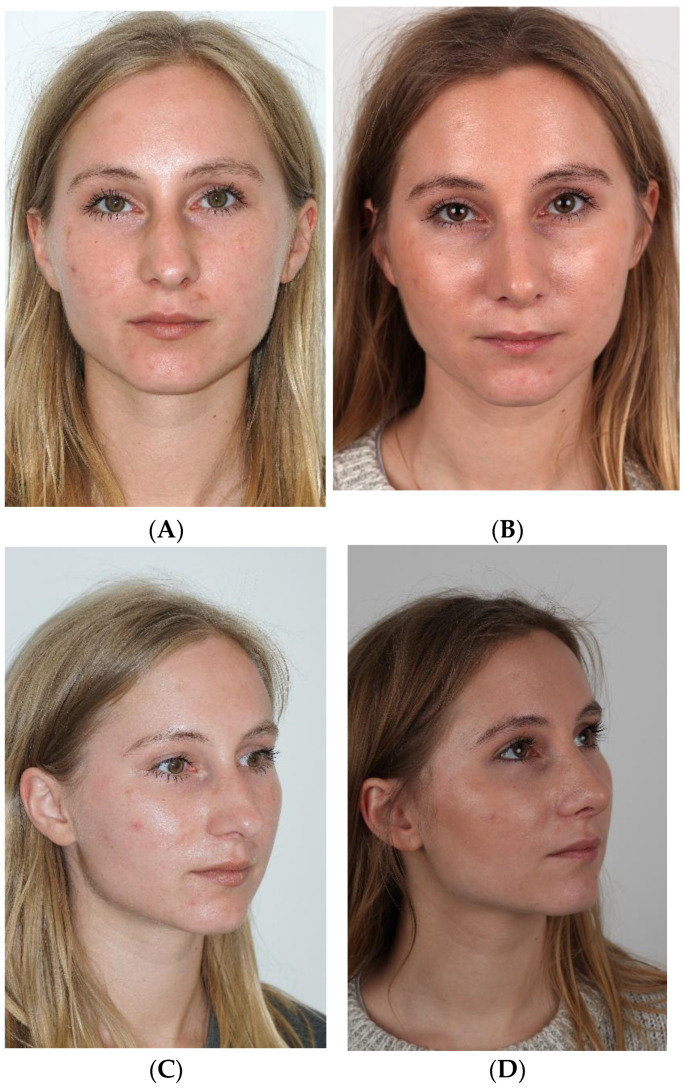

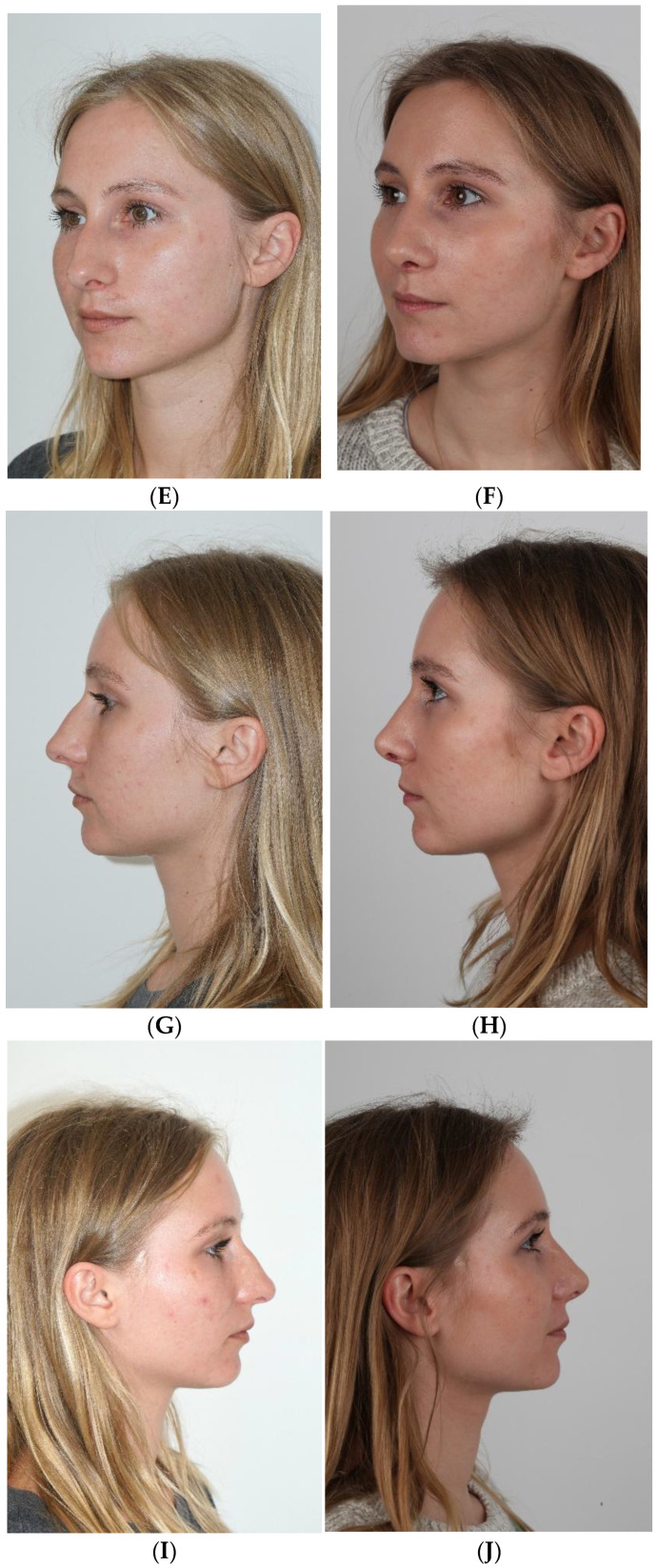
(**A**–**J**) Pre- (**A**,**C**,**E**,**G**,**I**) and postoperative (**B**,**D**,**F**,**H**,**J**) pictures of patient (PR).

Figure 4A–J and Figure 1A–J show cases treated using the PR technique.

## 4. Discussion

Rhinoplasty, as one of the most demanding procedures in facial plastic surgery, requires the surgeon not only to have excellent anatomical knowledge and high technical skills but also the ability to make often difficult, conscious intraoperative decisions and to communicate with the patient. The surgeon must simultaneously attend to the aesthetic and functional aspects of the nose because even a perfect anatomical outcome does not guarantee patient satisfaction if it does not meet their visual or respiratory expectations. In clinical practice, patients often focus primarily on the appearance of the nose, overlooking its respiratory function. As Kemal [12] points out, the aesthetic effect is a key factor determining postoperative satisfaction. Even if problems related to nasal obstruction are effectively resolved, dissatisfaction with the shape or proportions of the nose can lead to a negative assessment of the entire treatment. This means the perception of the operation’s success is subjective and largely depends on the patient’s expectations. For this reason, the use of objective, validated assessment tools such as the ROE, SCHNOS, or NOSE questionnaires, both before and after the operation, seems particularly important [1,4,13]. These tools not only enable the objectification of functional and aesthetic conditions but also facilitate better communication with the patient, the identification of their needs, and the monitoring of treatment effects. The introduction of such tools helps build realistic expectations, facilitates informed therapeutic decision-making processes, and increases satisfaction with the procedure’s outcomes.

Despite first emerging in the literature over a century ago, PR has recently taken centre stage at global plastic surgery conferences [14]. Since the publication of the article “The Preservation Rhinoplasty: A New Rhinoplasty Revolution” [15], there has been a growing emphasis on the sparing approach in rhinosurgery. It has been accepted as a basic principle that preserving the original dorsum of the nose and lowering it by intervening in the lower structures is better than resection with subsequent reconstruction and that the natural postoperative outcome is better than an artificially created nasal structure [16].

Furthermore, it is believed that leaving the natural dorsal attachments intact should produce a satisfactory aesthetic outcome, minimising surface irregularities [17]. Scientific publications indicate that PR is an excellent surgical method, often considered superior, especially for a specific group of patients [18]. According to the literature, the ideal nose for PR is one with a high bridge, a convex dorsum, a prominent anterior nasal septal angle (ANSA) with a narrow middle third of the dorsum, thin nostrils (known as tension nose), and, depending on the characteristics, a crooked nasal bridge. It is also the preferred technique for patients with thin nasal skin [18]. Sources also suggest that patients with features such as a low bridge, bridge irregularities, an ANSA below the rhinion, and a wide middle third may derive greater postoperative benefits from the SR technique. Patients with a very low bridge, a significantly enlarged middle third, a residual hump, and a drooping supratip area constitute a group in which SR is highly recommended [18,19]. To standardise the results, the above groups of patients were excluded from the study analysis. To obtain the most reliable results, patients with characteristics that allow both classic and conservative surgical methods were included in the study, without a clear advantage of either method. Furthermore, the size of the dorsal hump did not differ significantly between the groups.

During rhinoplasty, surgeons initially dissected the soft tissues in the subcutaneous plane; however, dissection is now performed mainly under the SMAS layer, where less vascularisation is expected. Despite the greater ease of tissue separation, dissection under the SMAS is associated with significant swelling and is often accompanied by numbness persisting after the procedure in the supratip area, scarring, hardening of the operated area, or thinning of the SSTE, as shown in Structure Rhinoplasty: Lessons Learned in 30 Years by Toriumi [15]. Subperichondrial–subperiosteal dissection (SSD), performed using the PR technique, results in less intraoperative bleeding, minimal swelling, a shorter healing period, a negligible feeling of numbness, less scar formation, and protection of the SSTE layer from long-term thinning [16].

The study compared patient outcomes (before and after surgery) using the ROE, SCHNOS, NOSE, and WHOQOL-BREF questionnaires.

Statistical analysis of the NOSE questionnaire showed an increase in patient satisfaction, regardless of surgical technique. At the same time, statistically significant differences were found in the SCHNOS-O and ROE parameters during the first and second postoperative follow-ups, respectively.

Patients in the SR group achieved worse scores than those in the PR group. Considering the SCHNOS-O domain, responsible for nasal function, a likely factor influencing the differences presented in the study could have been postoperative swelling, which, however, did not significantly affect satisfaction with the appearance of the external nose. Patients in the study groups showed improvement in the aesthetic component (Table 3 and Table 4). As proven in the works by Kosins et al. [20,21], “Comprehensive Diagnosis and Planning for the Difficult Rhinoplasty Patient: Applications in Ultrasonography and Treatment of the Soft-Tissue Envelope” and “Managing the Difficult Soft Tissue Envelope in Facial and Rhinoplasty Surgery”, when assessing patients qualified for rhinoplasty and after the procedure using ultrasonography, the dissection plane has a significant impact on the SSTE healing and postoperative swelling, including within the nasal cavity. After dissection under the SMAS, the dermis and soft tissues become swollen and significantly thickened, thereby decreasing the respiratory volume of the nasal cavities. Peak swelling is noticeable in the first month, followed by gradual reorganisation and final thinning of the soft tissue.

Studies of patients who underwent SSD show less swelling in the first month than with the previously described technique, reaching a steady state after approximately 3 months, with less soft-tissue thinning. Statistical analysis of the study results showed a statistically significant improvement after surgery (after 1 and 3 months) regardless of the method used. According to the analysis, patients reported the greatest dissatisfaction in the SCHNOS-O questionnaire before surgery, most likely related to nasal septal curvature and INV abnormalities. Improvement was gradually noticeable during subsequent visits, which may indicate a properly performed surgical procedure and a gradual reduction of swelling. It is worth noting that, despite differences in perceived improvement in nasal cavity function, in both cases, regardless of the type of surgery, an improvement in quality of life was observed across all domains (somatic, psychological, social, environmental, and self-assessment). This can be explained by studies of patients following rhinoplasty, which suggest that when the cosmetic effect of the surgery is satisfactory, patients may place less emphasis on functional aspects.

In the aesthetic component of the SR and PR groups, improvement was observed at each visit, regardless of the surgical technique used. Citing available sources, similar results have been obtained in studies evaluating PR postoperative improvement in long-term follow-up. Saban [22], analysing a total of 320 patients (286 women and 34 men, aged 13 to 71, with a mean age of 29), published a study entitled “Dorsal Preservation: The Push Down Technique Reassessed” [22], which showed postoperative satisfaction, covering an average follow-up period of 2 years and 5 months (ranging from 6 months to 5.5 years). Depending on individual pathologies and preoperative assessment, various PR techniques were used. The planned extent of hump reduction determined the method choice. The revision rate for the procedures performed was 3.4%. Importantly, none of the complications, such as deformation of the middle third, cerebrospinal fluid leakage, anosmia, or nasal obstruction, occurred in the study groups.

In turn, Kovacevic, in his paper “Die nasenrückenerhaltende ‘Dorsal-Preservation’-Septorhinoplastik” [23], described improvement in 205 PR patients with a revision rate of less than 10%. Atolini, in “Septum pyramidal adjustment and repositioning—a conservative and effective rhinoplasty technique” [24], analysed the postoperative outcomes of 153 patients (55 men and 98 women, with a mean age of 27.2). In the long-term evaluation, the revision rate was 8.5%. Rabaioli et al. [25] emphasised that even with minor functional problems after surgery, overall patient satisfaction remains high due to the positive aesthetic outcome. This means that achieving aesthetic improvement may outweigh functional concerns, especially if the preoperative motivation was driven by aesthetic considerations. However, it is worth noting that in some cases, unresolved functional problems may lead to dissatisfaction in the future, even if the aesthetic goals were initially achieved. For this reason, improvement should always be sought in every aspect, not only cosmetic but also functional [25,26,27].

Both PR and SR can be performed using a closed technique (intranasal approach) or an open technique (extranasal approach) [15]. As mentioned, PR and SR were performed via an extranasal approach, with subsequent ligament reconstruction using the PR technique to achieve the optimal postoperative outcome. Informed by contemporary concepts of rhinoplasty, authors such as Rollin K. Daniel et al. [28], Mauro Barone et al. [29], and Ali Y. Helmy et al. [30] recommend, based on their research (“The Nasal Ligaments and Tip Support in Rhinoplasty: An Anatomical Study” [28], “Reconstruction of Scroll and Pitanguy’s Ligaments in Open Rhinoplasty: A Controlled Randomised Study” [29], “Ligament Preservation in Open Rhinoplasty: Prospective Analysis” [30]), preserving the nasal ligaments and restoring their course in the event of transection. It is assumed that if part of the ligaments in this SLC complex remains intact during surgery, the function of the nose will be preserved with minimal risk of scar contracture [30]. In patients with scarring, abnormal SSTE thickness can often be observed as early as a few weeks, leading to deterioration in both the aesthetics and function of the nose [20,21]. Therefore, many techniques have been developed to preserve and/or restore severed nasal ligaments. For example, preserving the intact SLC complex by connecting subperichondrial dissection pockets between the lower lateral cartilages (LLC) and upper lateral cartilages (ULC) allows for the creation of a clear aesthetic line, closure of the dead space, and stabilisation of the INV [31,32]. In the case of reconstruction, one of the most frequently described examples in the literature is the reconstruction of Pitanguy’s midline ligament. During the procedure, the ligament is isolated and marked before cutting for later identification. At the end of the procedure, the ligament is repaired, pulling the soft tissue downwards and thereby reducing the dead space above the tip, allowing better repositioning of the soft tissues and stabilising the tip [29,30]. The authors suggest that ligament reconstruction/preservation is an alternative method for achieving projection and rotation of the tip without the use of classic columellar grafts or septal extension grafts.

## 5. Conclusions

Comparing PR with SR is an important element in assessing the effectiveness of modern surgical methods in nasal surgery. Both techniques differ in their assumptions, the extent of interference with anatomical structures, and the intraoperative strategy, which may affect aesthetic and functional outcomes as well as recovery time. Informed choice of surgical procedure, taking into account the individual needs of the patient, enables optimisation of treatment outcomes, reduction of the risk of complications, and increased postoperative satisfaction. In everyday clinical practice, patients, often influenced by information available on the internet, may place pressure on surgeons to select a specific technique based on expectations of faster or more visible early results. Therefore, understanding and objectively evaluating these early outcomes has practical value in patient counselling and in setting realistic expectations.

Analysis of follow-ups showed that PR patients had higher satisfaction levels in the early postoperative period. The effects of treatment increased gradually, reaching their peak at the second postoperative check-up. Before the procedure, patients’ quality of life and self-assessment were significantly lower. In contrast, after the operation, a significant improvement was noted, confirming the positive impact of surgical intervention on patients’ overall well-being. The results of the ROE, SCHNOS, and NOSE questionnaires showed similar statistical significance across both groups, suggesting comparable effectiveness of the methods, despite differences in surgical approach.

## Figures and Tables

**Table 1:** Questionnaire results in the analysed patient groups. Descriptive statistics along with the Shapiro–Wilk normality test in patients after PR.

	*M*	*Mdn*	*SD*	*Sk.*	*Kurt.*	*Min.*	*Max.*	*W*	*p*
ROE: measurement 1	1.2	1.2	0.7	0.5	−0.6	0.2	2.8	1	0.3
ROE: measurement 2	3.5	3.6	0.4	−0.4	−0.8	2.7	4	0.9	0.04
ROE: measurement 3	3.7	3.8	0.3	−1.5	3.2	2.7	4	0.9	<0.001
SCHNOS-O: measurement 1	3	3	1.3	−0.3	−1	0.8	5	0.9	0.1
SCHNOS-O: measurement 2	0.9	0.8	0.5	0.4	−0.3	0	2	1	0.1
SCHNOS-O: measurement 3	0.5	0.5	0.4	0.9	0.6	0	1.5	0.9	0.01
SCHNOS-C: measurement 1	3.6	3.8	1.3	−1.5	1.8	0	5	0.8	<0.001
SCHNOS-C: measurement 2	0.7	0.4	0.9	1.8	2.5	0	3.7	0.8	<0.001
SCHNOS-C: measurement 3	0.6	0.3	0.8	1.5	1.3	0	2.5	0.8	<0.001
Total SCHNOS: measurement 1	3.4	3.4	1	−1.4	2.9	0.4	5	0.9	<0.001
Total SCHNOS: measurement 2	0.8	0.6	0.6	1.2	0.5	0	2.4	0.8	<0.001
Total SCHNOS: measurement 3	0.6	0.3	0.5	1.3	0.5	0	1.9	0.8	<0.001
Quality of life—somatic: measurement 1	26	26	3.5	−0.01	−1.2	20	32	1	0.1
Quality of life—somatic: measurement 2	30	30	2.8	−0.1	−1	25	34	0.9	0.1
Quality of life—psychological: measurement 1	23	23	3.2	−0.6	0.4	15	29	1	0.2
Quality of life—psychological: measurement 2	26	26	2.2	−0.2	−0.6	21	30	1	0.3
Quality of life—social: measurement 1	12	12	2.3	−0.3	−0.2	6	15	0.9	0.02
Quality of life—social: measurement 2	13	14	1.7	−0.5	−1.1	10	15	0.9	0.001
Quality of life—environmental: measurement 1	32	32	3.4	0.2	−0.9	26	38	0.9	0.1
Quality of life—environmental: measurement 2	34	34	3.3	−0.7	−0.3	26	38	0.9	0.02
Self-assessment: measurement 1	33	34	4.7	−0.5	−0.3	23	40	0.9	0.1
Self-assessment: measurement 2	37	37	3.4	−0.9	−0.2	29	40	0.9	<0.001
NOSE: measurement 1	3.3	3.4	0.8	−0.5	0	1.2	4.4	1	0.2
NOSE: measurement 2	1.6	1.6	0.5	1.1	1.5	0.8	2.8	0.9	0.001

measurement 1—assessment before surgery; measurement 2—assessment one month after surgery; measurement 3—assessment three months after surgery.

**Table 2:** Questionnaire results in the analysed patient groups. Descriptive statistics along with the Shapiro–Wilk normality test in patients after SR.

	*M*	*Mdn*	*SD*	*Sk.*	*Kurt.*	*Min.*	*Max.*	*W*	*p*
ROE: measurement 1	1.1	1.2	0.6	−0.03	−1.1	0.2	2.2	1	0.1
ROE: measurement 2	3.4	3.3	0.5	−1.4	2.2	1.7	4	0.9	0.003
ROE: measurement 3	3.5	3.7	0.4	−1.1	0.9	2.3	4	0.9	<0.001
SCHNOS-O: measurement 1	3.4	3.3	1.1	−0.5	−0.2	1	5	0.9	0.1
SCHNOS-O: measurement 2	1.3	1.3	0.7	0.5	0.5	0.3	3.3	1	0.3
SCHNOS-O: measurement 3	0.6	0.5	0.5	0.5	−0.8	0	1.8	0.9	0.01
SCHNOS-C: measurement 1	3.9	4	0.8	−0.6	0.1	1.8	5	0.9	0.04
SCHNOS-C: measurement 2	0.8	0.5	0.9	1.9	3.4	0	3.7	0.8	<0.001
SCHNOS-C: measurement 3	0.6	0.3	0.7	2.0	3.6	0	2.8	0.8	<0.001
Total SCHNOS: measurement 1	3.7	3.7	0.6	0.4	−0.2	2.4	5	1	0.3
Total SCHNOS: measurement 2	1	0.8	0.7	1.7	2.8	0.1	3.3	0.9	<0.001
Total SCHNOS: measurement 3	0.6	0.4	0.5	1.5	1.9	0	2.2	0.8	<0.001
Quality of life—somatic: measurement 1	27	28	3.8	−0.6	−0.3	17	32	0.9	0.1
Quality of life—somatic: measurement 2	29	30	3.7	−0.4	−0.9	21	34	0.9	0.03
Quality of life—psychological: measurement 1	23	23	3.3	−0.7	0.7	14	29	1	0.3
Quality of life—psychological: measurement 2	26	26	2.9	−0.5	−0.6	19	30	0.9	0.1
Quality of life—social: measurement 1	12	12	2.4	−0.6	0.03	6	15	0.9	0.01
Quality of life—social: measurement 2	13	13	2.1	−0.4	−1.1	9	15	0.9	0.002
Quality of life—environmental: measurement 1	32	32	3.3	−0.9	2.2	21	38	1	0.1
Quality of life—environmental: measurement 2	33	34	3.4	−1	0.5	25	38	0.9	0.02
Self-assessment: measurement 1	33	32	4.4	−0.4	−0.1	23	40	0.9	0.03
Self-assessment: measurement 2	35	37	3.6	−0.3	−1.5	30	40	0.9	<0.001
NOSE: measurement 1	3.1	3.2	1	−0.1	−0.8	1.6	4.6	1	0.4
NOSE: measurement 2	1.5	1.4	0.5	1.9	5	1	3.6	0.8	<0.001

**Table 3:** Comparison of results for patients operated on using the preservative (PR) and classic (SR) methods.

	PR (n = 36)			SR (n = 39)					
Dependent Variable	Average Rank	*Mdn*	*IQR*	Average Rank	*Mdn*	*IQR*	*Z*	*p*	*R*
ROE: measurement 1	38	1.2	1.1	38	1.2	1	−0.1	0.9	0.01
ROE: measurement 2	40	3.6	0.7	36	3.3	0.7	−0.8	0.4	0.1
ROE: measurement 3	43	3.8	0.3	33	3.7	0.5	−2.1	**0.04**	0.2
SCHNOS-O: measurement 1	35	3	1.9	41	3.3	1.5	−1.1	0.3	0.1
SCHNOS-O: measurement 2	31	0.8	0.8	45	1.3	1	−2.7	**0.01**	0.3
SCHNOS-O: measurement 3	37	0.5	0.5	39	0.5	0.8	−0.5	0.6	0.1
SCHNOS-C: measurement 1	37	3.8	1.4	39	4	1.3	−0.3	0.8	0.03
SCHNOS-C: measurement 2	36	0.4	1	40	0.5	0.8	−0.6	0.6	0.1
SCHNOS-C: measurement 3	37	0.3	1	39	0.3	0.5	−0.3	0.8	0.03
Total SCHNOS: measurement 1	36	3.4	1	40	3.7	0.8	−0.8	0.4	0.1
Total SCHNOS: measurement 2	33	0.6	0.7	42	0.8	0.8	−1.8	0.1	0.2
Total SCHNOS: measurement 3	38	0.3	0.6	39	0.4	0.6	−0.2	0.8	0.02
NOSE: measurement 1	41	3.4	1.2	36	3.2	1.2	−1	0.3	0.1
NOSE: measurement 2	39	1.6	0.6	35	1.4	0.6	−0.7	0.5	0.1

**Table 4:** Comparison of ROE and SCHNOS scores between measurements in patients operated on using the PR and SR methods.

	Measurement 1		Measurement 2		Measurement 3				
	*Mdn*	*IQR*	*Mdn*	*IQR*	*Mdn*	*IQR*	χ^2^	*p*	Post hoc ^a^
**PR**									
ROE	1.2	1.1	3.6	0.7	3.8	0.3	67	<0.001	1<2; 1<3;
SCHNOS-O	3	1.9	0.8	0.8	0.5	0.5	63	<0.001	1>2; 1>3; 2>3
SCHNOS-C	3.8	1.4	0.4	1	0.3	1	64	<0.001	1>2; 1>3;
Total SCHNOS	3.4	1	0.6	0.7	0.3	0.6	66	<0.001	1>2; 1>3; 2>3
**SR**									
ROE	1.2	1	3.3	0.7	3.7	0.5	72	<0.001	1<2; 1<3;
SCHNOS-O	3.3	1.5	1.3	1	0.5	0.8	75	<0.001	1>2; 1>3; 2>3
SCHNOS-C	4	1.3	0.5	0.8	0.3	0.5	72	<0.001	1>2; 1>3;
Total SCHNOS	3.7	0.8	0.8	0.8	0.4	0.6	7.5	<0.001	1>2; 1>3; 2>3

^a^—a post hoc analysis using Dunn’s test with Bonferroni correction. The table shows the differences between measurements, along with the direction of statistically significant differences (*p* < 0.05).

**Table 5:** Comparison of NOSE scores in two measurements in patients operated on using the PR and SR methods.

	Measurement 1		Measurement 2				
Dependent Variable	*Mdn*	*IQR*	*Mdn*	*IQR*	*Z*	*p*	*r*
NOSE–PR	3.4	1.2	1.6	0.6	−5.2	**<0.001**	0.6
NOSE–SR	3.2	1.2	1.4	0.6	−5.3	**<0.001**	0.6

**Table 6:** Summary of scores taking into account quality of life, self-assessment, and type of surgery.

Model	Effects	*F*	*df*	*p*	η_p_^2^
Model 1	Quality of life—somatic	193	1;63	<0.001	0.75
	Surgery type	0.01	1;63	0.99	<0.01
	Interaction	2.4	1;63	0.13	0.04
Model 2	Quality of life—psychological	159	1;63	<0.001	0.7
	Surgery type	0.01	1;63	0.95	<0.01
	Interaction	0.5	1;63	0.5	0.01
Model 3	Quality of life—social	36	1;63	<0.001	0.4
	Surgery type	0.1	1;63	0.8	<0.01
	Interaction	1.9	1;63	0.2	0.03
Model 4	Quality of life—environmental	41	1;63	<0.001	0.4
	Surgery type	0.01	1;63	0.9	<0.01
	Interaction	1	1;63	0.3	0.02
Model 5	Self-assessment	96	1;73	<0.001	0.6
	Surgery type	0.6	1;73	0.4	0.01
	Interaction	1.9	1;73	0.2	0.03

## Data Availability

The data presented in this study are available upon request from the corresponding author.
